# Quantifying genetic differences between exported dairy bull calves and those sold for domestic beef production

**DOI:** 10.3168/jdsc.2021-0105

**Published:** 2021-09-13

**Authors:** D.P. Berry, S.C. Ring, A.J. Twomey

**Affiliations:** 1 Teagasc, Animal & Grassland Research and Innovation Centre, Moorepark, Fermoy P61 P302, Co. Cork, Ireland; 2 Irish Cattle Breeding Federation, Highfield House, Shinagh, Bandon P72 X050, Co. Cork, Ireland

## Abstract

Selection bias is introduced when selection among individuals exists but the information used to inform that selection decision is not considered in downstream genetic evaluations. Genetic evaluations are undertaken in several countries for carcass-related metrics in prime cattle; no consideration is generally taken for animals that are harvested at a younger age for veal production and thus do not express the prime carcass phenotype. Although no veal industry exists in Ireland, dairy calves are routinely exported to continental Europe for veal production. The objective of the present study, based on a cross-sectional analysis of calf export data, was to determine quantitatively if genetic variability exists in whether purchased dairy-bred bull calves are immediately exported or retained within the country for domestic production. Also of interest was whether such a genetic difference was associated with differences in carcass weight, conformation score, and fat score in prime cattle relatives. Editing criteria were imposed to consider only Holstein-Friesian bull calves. Post-editing, the fate of 43,890 Holstein-Friesian bull calves (<100 d of age) was available; variance components for the binary phenotype (sold for export or not) were estimated using both linear and threshold animal models, and genetic correlations with carcass traits from 56,366 prime cattle were estimated. The heritability (standard error) of whether or not a calf was exported was 0.04 (0.01) on the linear scale and 0.07 (0.02) on the threshold scale. Although no explicit maternal genetic effect was detected, the proportion of the phenotypic variance due to maternal effects was 0.03 to 0.07. The genetic correlation (standard error) between the export phenotype with carcass weight, conformation score [scale 1 (poor) to 15 (excellent)], and fat score [scale 1 (thin) to 15 (fat)] in prime cattle was 0.002 (0.12), −0.25 (0.12), and −0.32 (0.11), respectively. The low heritability of the calf export phenotype and lack of a strong genetic correlation with carcass metrics suggest that other calf features might be greater determinants of the eventual fate of the calf. Accounting for the export phenotype in genetic evaluations of carcass traits in prime cattle had a negligible effect on the estimated breeding values for carcass merit.

## Highlights

• Heritability of whether a Holstein-Friesian bull calf was purchased for export was 0.04.
• The probability of a bull calf being purchased for export was greatest when the calf was 2 wk of age.
• Dam parity did not influence the likelihood of a bull calf being purchased for export.
• Accounting for exported calves in genetic evaluations had a negligible impact on carcass genetic evaluations.

## Graphical Abstract

**Figure:**
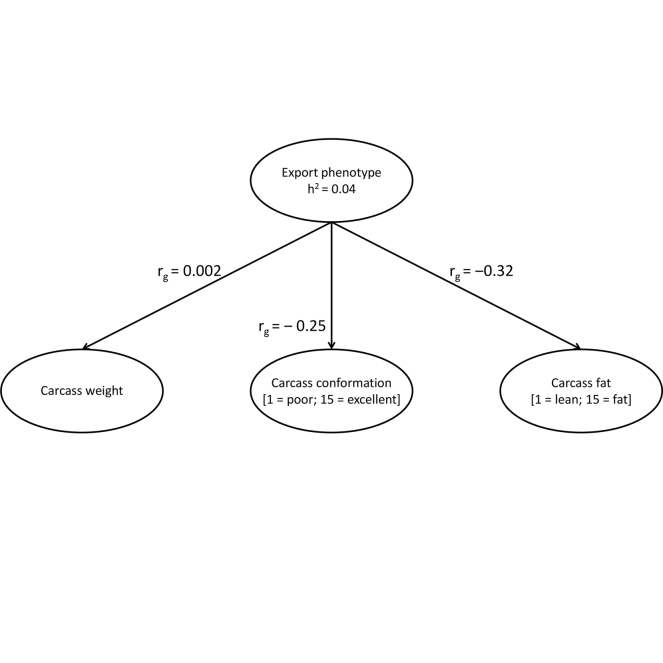
**Summary:** While many progeny from dairy herds are reared domestically for eventual processing as prime cattle, many are processed as calves to supply the veal market. Although no veal industry exists in Ireland, a selection of dairyborn calves traded privately or in livestock auctions are purchased for export to continental Europe for veal production. This study demonstrated that genetic variability does exist in which Holstein-Friesian male calves are chosen for export versus retained domestically for eventual slaughter at an older age (i.e., >1 yr of age); in fact, 4% of the variability of whether a purchased bull calf was exported could be attributable to underlying genetic differences. No strong genetic correlation was evident between whether a calf was exported (for veal) and the expression of carcass merit in prime cattle relatives. Moreover, not accounting for these exported calves in national carcass genetic evaluations is not a cause of concern for the potential introduction of bias.

Several potential markets exist for dairy-bred calves (Berry, 2021). The majority of dairy heifer calves graduate to the mature dairy herd (Kelleher et al., 2016), while some dairy × beef heifers graduate into the mature beef herd (Berry et al., 2006). Meat products from the resulting surplus calves that do not succumb to premature death enter the food chain. These calves can be processed at a very young age (i.e., bobby calves), processed for veal, or processed as “prime cattle,” which are slaughtered at >1 yr of age never having parented an offspring (Berry, 2021). Dairy farmers generally do not breed for a given market; most dairy farmers sell their calves at a few weeks of age at public livestock auctions (Mc Hugh et al., 2010; Dunne et al., 2021), and the eventual fate of the calves is determined by, among others, the purchasers on the day (e.g., calf exporters or Irish beef producers). Genetic evaluations exist for carcass-related traits of prime cattle (i.e., carcass weight, conformation score, fat score, primal cuts) in several countries, many of which originate from dairy herds initially (Pabiou et al., 2012; Englishby et al., 2016; Pritchard et al., 2021). No consideration in the genetic evaluation, however, is generally taken of animals that do not express these phenotypes (e.g., those processed for veal). If preselection of calves for the veal versus the prime market exists based on some features correlated with eventual carcass merit, then not considering this market in the genetic evaluation of prime cattle could bias these evaluations.

The objective of the present study was to use interlocation animal movement data from young Holstein-Friesian bull calves to quantify whether genetic differences exist between calves exported for veal production and those retained for domestic production of prime carcasses. Although Ireland does not operate veal production to any great extent, it does export tens of thousands of dairy bull calves annually for veal production (Department of Agriculture, Food and the Marine, 2020). In 2019, for example, 114,063 dairy male calves <6 wk of age were exported from Ireland. Within Ireland, the movement of all animals is recorded, including the location of origin and destination. Carcass information on all prime animals slaughtered in Ireland is also available. Hence, this large national data set provides a rich information source by which to quantify the genetic parameters for dairy bull calf destination. The impact of accounting for calves being diverted for veal production on the genetic evaluation of prime cattle carcass merit can also be quantified.

All data were extracted from the Irish Cattle Breeding Federation (http://www.icbf.com) database. It is legally required in Ireland to record the birth, death, and movement of all animals between different holdings, including those exported. Information was available on 1,639,224 bull calves sired by multiple breeds exported from Ireland between 7 and 100 d of age over the 15-yr period from 2006 to 2020. Information available included both the date and herd of birth, as well as the date of export of all calves, along with an anonymous identifier of the exporter. Only data from 17 exporters that exported more than 5,000 calves over the 15-yr period were retained. Added to the data set were movement records for contemporary calves from the same herds that were not exported. Calves that were not exported were only considered in the analysis if their age on the date of sale was within the range of ages purchased by the exporter(s) on that day.

The sire and dam of all calves had to be known and all were of the Holstein-Friesian breed; a total of 715,838 Holstein-Friesian bull calves remained. Additionally, because not all calves are immediately exported, only bull calves that were exported within 1 wk of being purchased from their herd of birth were retained; 665,054 animals remained. Contemporary group was defined as herd (of birth) by date of purchase, including calves that were exported as well as their contemporaries that were not. Only contemporary groups with at least 15 calves were retained for variance component estimation. Following all edits, information on whether or not a calf was purchased for export was available on 43,890 calves from 2,324 contemporary groups originating from 1,201 dairy herds.

Carcass weight and conformation and fat scores of prime cattle slaughtered in Ireland were available over the same time period (i.e., 2006 to 2020, inclusive). Carcass conformation and carcass fat are both recorded using the 15-point EUROP classification systems (Englishby et al., 2016). Scores of 1 for carcass conformation and fat represent poor conformation and low fat cover, respectively, whereas scores of 15 represent the opposite (Englishby et al., 2016). Edits imposed for quality control of the carcass data were described in detail elsewhere (Twomey et al., 2020); the only addition was that only Holstein-Friesian cattle were retained. Carcass records were discarded from animals that resided in more than 3 herds during their lifetime or were present in the herd they were slaughtered from for <100 d before slaughter. All animals had to have a known sire and had to be born in a dairy herd, as defined by Ring et al. (2018). Contemporary group was defined as herd-sex-year-season of slaughter (Twomey et al., 2020); to enable the estimation of variance components, a random sample of contemporary groups was retained. The edited data set consisted of carcass trait information on 56,366 cattle from 6,687 contemporary groups. Of the prime cattle with carcass information, 28,404 had at least one parental half-sib with an export phenotype; of the calves with an export phenotype, 33,642 had at least one parental half-sib prime animal with carcass information. No exported animal had a carcass phenotype but 2,507 animals with an export phenotype (i.e., not exported) had a carcass phenotype.

Variance components for whether or not a calf was purchased for export were estimated using both an animal linear mixed model and an animal threshold model in Asreml (Gilmour et al., 2009). Preliminary analyses revealed a maternal environmental contribution to the variability in whether or not a calf was purchased for export; no maternal genetic variance was detected. Therefore, the model fitted was

*Y_ijklmn_* = *age_k_* + *dam_parity_l_* + *het_m_* + *CG_n_* + *a_i_* + *dam_j_* + *e_ijklmn_*,

where *Y_ijklmn_* is the binary outcome of whether or not calf *i* from dam *j* was purchased for export; *age_k_* is the age of the calf in weeks (*k* = 1–13 wk) when purchased; *dam_parity_l_* represents the parity of the dam (*l* = 1, 2, 3, 4, 5+); *het_m_* is the heterosis coefficient *m* of calf *i* as a continuous variable; *CG_n_* is the contemporary group of herd-date of sale (*n* = 2,324); *a_i_* is the random additive genetic effect of calf *i*; $N\left(0,A\sigma_{a}^{2}\right),$ where $\sigma_{a}^{2}$ is the additive genetic variance and **A** is the numerator relationship matrix; *dam_j_* represents the dam *j* of animal *i* $N\left(0,I\sigma_{dam}^{2}\right),$ with $\sigma_{dam}^{2}$ representing the dam variance and **I** an identity matrix; and *e_ijklmn_* represents the residual term where $N\left(0,I\sigma_{e}^{2}\right),$ with $\sigma_{e}^{2}$ representing the residual variance. The pedigree of all animals was traced back to the founder population; the pedigree file consisted of 304,062 unique animals.

Genetic covariances between the exported phenotype with each of the 3 carcass traits were estimated using a series of sire linear mixed models. The model fitted to the export phenotype was as previously described, except that the animal additive genetic effect was replaced with a sire additive genetic effect. A total of 11,006 sires were represented in the bivariate data set, with a mean of 8 progeny per sire; 1,128 sires had progeny with both an export and a carcass phenotype. The model fitted to the carcass traits was

*Y_ilmn_* = *dam_parity_l_* + *het_m_* + *CG_n_* + *s_i_* + *e_ilmn_*,

where *Y_ilmn_* is carcass weight, conformation score, or fat score; *dam_parity_l_* represents the parity of the dam (*l* = 1, 2, 3, 4, 5+); *het_m_* is the heterosis coefficient *m* of the animal as a continuous variable; *CG_n_* is the contemporary group of slaughter (*n* = 6,688); *s_i_* is the random additive genetic effect of sire *i*, where $N\left(0,A\sigma_{s}^{2}\right),$ with $\sigma_{s}^{2}$ representing the sire genetic variance and **A** the numerator relationship matrix; and *e_ilmn_* represents the residual term where $N\left(0,I\sigma_{e}^{2}\right),$ with $\sigma_{e}^{2}$ representing the residual variance and **I** an identity matrix.

Following the estimation of variance components, genetic evaluations for the 3 carcass traits were undertaken using a multi-trait linear mixed model that included either the 3 carcass traits alone or the 3 carcass traits with the export phenotype. To increase the size of the data set for the genetic evaluations, the previously described edit of at least 15 animals per contemporary group for the export phenotype was relaxed to 5, and no random sampling of contemporary groups for the carcass traits was undertaken. In total, 1,109,068 animals were included in the evaluations, of which 43,969 had an export phenotype and 1,067,606 had a carcass phenotype; the pedigree of 2,977,947 animals was used. The statistical model used in the genetic evaluations was that already defined in the present study for the estimation of variance components. The (co)variance components for the export phenotype used in the genetic evaluation were those estimated in the present study, with the (co)variance components among the carcass traits being those actually used in the respective national genetic evaluation. The EBV from both evaluations were rebased to a common base, and the EBVs of the 46,631 sires with at least one progeny in the data set were compared.

The mean age of the calves purchased for export was 27 d with a standard deviation (**SD**) of 12.6 d (Figure 1); the mean age of the contemporary calves not sold for export was 29 d (SD = 17.4 d). The odds of being purchased for export by week of age when sold is shown in Figure 1. The odds increased to a peak at 2 wk of age (i.e., 14 to 20 d of age; 69% of calves sold were exported) with an odds ratio (95% CI) of 2.1 (1.82–2.34) relative to being sold at 1 wk of age (i.e., 7 to 13 d of age; 46% of calves sold being exported); the odds reduced consistently thereafter. The odds of a calf being purchased for export at 2 wk of age was almost 4 times (i.e., 3.9) that of a 5-wk-old calf. Calf exporters from Ireland desire a calf of approximately 50 kg in live weight. In developing a decision support tool to predict what age a dairy bull calf is likely to reach 50 kg of live weight, Dunne et al. (2021) cited a mean age of 17 d (from a mature dam) to achieve 50 kg of live weight, albeit with an SD of 5.2 d. Hence, both studies agree that most dairy calves are purchased for export at approximately 2 wk of age. The Netherlands is the main market for these calves (Department of Agriculture, Food and the Marine, 2020), where they are processed for veal at approximately 27 wk of age.

**Figure 1:**
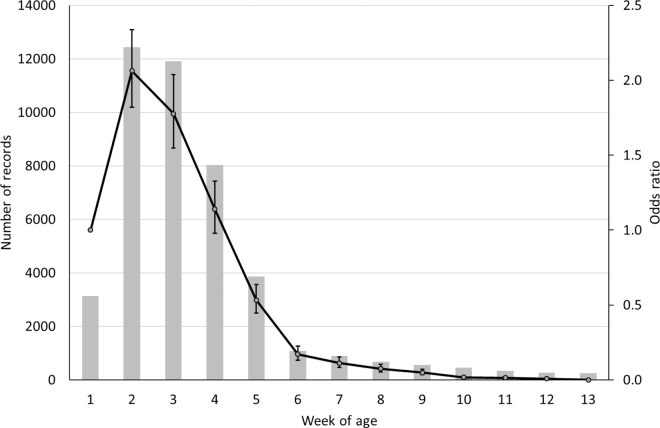
Number of records (histogram) for each week of age and odds ratio (line) of being purchased for export relative to calves sold at 1 wk of age; error bars indicate 95% CI of odds ratios.

The proportion of calves exported per contemporary group averaged 0.66, varying from 0.03 to 0.95. Of the 720 sires with at least 10 progeny in the edited data set, the mean proportion of progeny exported per sire was 0.64 (SD = 0.20). Dam parity was not associated (*P* = 0.58) with the likelihood of a Holstein-Friesian bull calf being sold for export. Calves in the edited data set were, on average, 72% Holstein with a mean heterosis coefficient of 0.41. The heterosis coefficient (i.e., between Holstein and Friesian) was negatively associated (*P* = 0.04) with the odds of the calf being sold for export; the logit of the odds (SE) on heterosis coefficient was −0.27 (0.13), implying that exporters sought more purebred Holstein or Friesian animals.

The heritability of whether or not a Holstein-Friesian bull calf was sold for export was low, irrespective of whether it was estimated using a linear model (0.04; SE = 0.01) or a threshold model (0.07; SE = 0.02); the respective genetic SD were 0.08 and 0.52. The proportion of the phenotypic variance attributable to the dam effect was 0.07 (SE = 0.02) and 0.03 (SE = 0.02) for the linear and threshold models, respectively. This equates to a phenotypic SD estimated using the linear model of 0.38, which is close to the expected value of 0.48 $\left(i.e.,\sqrt{pq}\right),$ given that 64% (i.e., p = 0.64; q = 0.36) of the calves in the edited data set were exported.

Given the lack of any previous analysis of such an export phenotype in calves, there was no expectation as to what the heritability of the fate of purchased dairy bull calves should be. Heavier calves should be more desirable by purchasers, and both growth rate and liveweight in calves tend to be moderately heritable (0.41 to 0.80; Brotherstone et al., 2007). Furthermore, Mc Hugh et al. (2011), in an analysis of 9,545 calves sold at auction <3 wk of age, reported a heritability of 0.34 for calf price. Moreover, the (visible) health status of the calf is expected to be important when deciding on whether to purchase a calf. The heritability of dairy calf health or wellness traits, such as respiratory disease or scours, has been reported to be generally ≤0.06 (Henderson et al., 2011; Gonzalez-Peña et al., 2019). Calf health status, especially that linked with a contagious pathogen, is expected to be particularly important for calves purchased for export. This is because the purchased calves will be joined with other calves (and thus exposed to other diseases) and expected to travel a long distance to their final location, where they could be mixed with more calves. Calf phenotypic health status, as well as being under direct genetic control (Henderson et al., 2011; Gonzalez-Peña et al., 2019), is influenced by the intake of a sufficient quantity of immunoglobulins, especially IgG, soon after birth (Robison et al., 1988). Although the features of the calf can influence its ability to absorb immunoglobulins from colostrum, the quantity and quality of immunoglobulins in the colostrum itself is also under genetic control (Conneely et al., 2013). Although an effect of dam on whether a calf was purchased for export was detected in the present study, no maternal genetic contribution to this dam effect was evident; the 43,890 calves with an export phenotype included in the analysis were the progeny of 41,658 dams. Whether or not a calf was purchased for export could be due to the contribution of dam factors other than colostrum quality. Overall, the fact that heritability was low for whether a purchased calf was exported suggests that the (perceived) health status or the veal potential of the calf was more important than the morphological characteristics of the calf per se.

The genetic correlations (SE) between the export phenotype and carcass weight, conformation score, and fat score in prime cattle were 0.002 (0.12), −0.25 (0.12), and −0.32 (0.11), respectively, indicating that progeny genetically predisposed to poorer conformation and leaner carcasses (assessed as prime cattle) were more likely to be purchased for export than retained for domestic use; no association with genetic merit for carcass weight was obvious. The lack of any correlation with animal size, as reflected by carcass weight, further substantiates the notion that animal features other than morphology, such as health and appearance, are greater determinants of whether an animal is exported. Moreover, the main differences likely to exist between the export or retention markets are that purchasers for the export market will purchase a large number of calves with the end market being veal; hence, such purchasers are very sensitive to calf price. The purchasers of calves for the domestic market are (predominantly) Irish farmers, mainly beef farmers. These purchasers are less sensitive to the individual unit price of calves, and the results from the current study confirm anecdotal evidence that Irish beef farmers actively seek animals with better (carcass) conformation, which will be expressed at slaughter as mature animals. Connolly et al. (2016) reported a 1.08-unit increase in phenotypic carcass conformation score per unit increase in EBV for carcass conformation. Berry et al. (2019) derived an economic value for carcass conformation of €17.58; hence, calves expected to generate more-conformed carcasses as prime cattle are likely to fetch a higher price. Information is sparse on whether conformation expressed as a prime animal is expressed to the same extent as a veal calf (van der Werf et al., 1998; Croué et al., 2017); both van der Werf et al. (1998) and Croué et al. (2017) reported genetic correlations between carcass traits expressed in prime cattle and the same carcass trait in veal calves of between 0.43 and 0.70. In the generation of a breeding index for veal production in the Netherlands, van der Werf et al. (1998) documented relative emphases of just 15% and 1% on carcass conformation and fat, respectively; growth rate and meat color represented 54% and 30% of the index, respectively. Hence, although conformation for veal production is important, it is not considered to be (in breeding programs, at least) as important as potential growth rate.

Many populations undertake genetic evaluations for carcass traits in prime cattle (Boukha et al., 2011; Pabiou et al., 2012; Pritchard et al., 2021), although those considering dairy breeds separately are fewer (Pritchard et al., 2021). Genetic evaluations generally include information on heifers, bulls, and steers slaughtered over 1 yr of age, often following an intensive finishing period. However, not all cattle born in dairy herds actually express the carcass phenotype; some animals die prematurely (Ring et al., 2019), others may become replacements in dairy (Kelleher et al., 2016) or beef (Berry et al., 2006) herds, with another cohort processed as bobby calves (Berry, 2021) or for veal production (Sans and de Fontguyon, 2009). In 2009, 40% of bull calves from the European Union dairy herd were processed as veal (Sans and de Fontguyon, 2009), although this does vary by country (Sans and de Fontguyon, 2009) and not all these countries have a genetic evaluation for carcass merit in prime cattle. In Ireland, between 2015 and 2019 (inclusive), a total of 420,049 bull calves <6 wk of age sired by a dairy bull were exported to another jurisdiction; annually, this represents 14 to 28% of dairy-sired bull calves born in Ireland. Hence, given that few dairy-bred heifers are processed for meat production, the proportion of dairy animals that do not express the eventual carcass phenotype as a prime animal can be considerable.

The existence of heritable genetic variability for the export phenotype indicates that breeding for calves more suited for the export market is possible; however, consideration needs to be given to the existence of such a market in the future, especially in light of sexed semen. Such a strategy is also important given the negative genetic correlation between the export phenotype and both carcass conformation and fat score. Carcass conformation in prime cattle has a positive economic value (Berry et al., 2019) and, even though most dairy animals are not processed as prime cattle, there is a growing beef-on-dairy market globally (Berry, 2021) as well as a considerable transfer of germplasm from the dairy to the beef herd in Ireland (Berry et al., 2006). Hence, erosion of carcass conformation in the pursuit of more suitable Holstein-Friesian calves for export could have unfavorable downstream ramifications. A more sensible approach would be to begin the process of generating the necessary data and associated systems to derive accurate genetic evaluations for calf health.

Given the observed (weak) genetic correlations between the export phenotype and carcass traits, complicating current genetic evaluations models for carcass traits in prime animals by including a correlated trait reflecting the export phenotype was not expected to deliver much benefit in precision of genetic evaluations. Although the export phenotype is measured very early in life and has a genetic correlation of −0.32 to −0.25 with carcass fat and conformation, the low heritability of the export phenotype implies little benefit as an early predictor, especially because the carcass traits are not sex-linked and tend to be measured early in life. In fact, the correlation between the EBVs among the carcass traits with or without accounting for the export phenotype was 1.0000 when sires with >20 carcass records were considered and 1.000 (carcass weight), 0.9985 (carcass conformation), and 0.9983 (carcass fat) when limited to sires with <20 progeny with carcass data and where more than half of their progeny had been exported. The regression of the difference in EBV for each carcass trait depending on whether the export phenotype was considered (i.e., EBV without considering the export phenotype minus EBV considering the export phenotype) on the proportion of calves per sire exported was 0.006 (SE = 0.001), 0.014 (SE = 0.001), and 0.012 (SE = 0.001) for carcass weight, conformation score, and fat score; these did not differ (*P* > 0.05) by the number of progeny with carcass records per sire. Hence, considering the export phenotype in a multi-trait genetic evaluation of carcass traits in prime cattle had negligible effect on the EBV.
